# Cerebral Melanoma Metastases: A Critical Review on Diagnostic Methods and Therapeutic Options

**DOI:** 10.5402/2011/276908

**Published:** 2011-05-25

**Authors:** Carlos R. Goulart, Tobias Alecio Mattei, Ricardo Ramina

**Affiliations:** Neurosurgery Department, Instituto de Neurologia de Curitiba, Jeremias Maciel Perretto Street, 300 Ecoville, Curitiba, PR 81210-310, Brazil

## Abstract

Malignant melanoma represents the third most common cause for cerebral metastases after breast and lung cancer. Central nervous system (CNS) metastases occur in 10 to 40% of patients with melanoma. Most of the symptoms of CNS melanoma metastases are unspecific and depend on localization of the lesion. All patients with new neurological signs and a previous primary melanoma lesion must be investigated. Although primary diagnosis may rely on computed tomography scan, magnetic resonance images are usually used in order to study more precisely the characteristics of the lesions in and to embase the surgical plan. Other possible complementary exams are: positron emission tomography, iofetamine cintilography, immunohistochemistry of liquor, monoclonal antibody immunocytology, optical coherence tomography, and transcriptase-polymerase chain reaction. Treatment procedures are indicated based on patient clinical status, presence of unique or multiple lesions, and family agreement. Often surgery, radiosurgery, whole brain radiotherapy, and chemotherapy are combined in order to obtain longer remissions and optimal symptom relieve. Corticoids may be also useful in those cases that present with remarkable peritumoral edema and important mass effect. Despite of the advance in therapeutic options, prognosis for patients with melanoma brain metastases remains poor with a median survival time of six months after diagnosis.

## 1. Introduction

 Metastatic spread of tumor cells detached from melanoma into the central nervous system (CNS) occurs haematogenically since lymphatic drainage is absent in the brain [1]. The blood-brain barrier is usually intact in metastases that are smaller than 0.25 mm in diameter [2]. 

The cells from brain metastases show a slower growth rate and exhibit lower metastatic potential than cells from visceral metastases, indicating that brain metastases do not necessarily represent the end stage in the metastatical cascade. Rather, brain metastases are likely to originate from a unique subpopulation of cells within the primary neoplasm [2]. 

## 2. Classification

Some authors found an association between the size of the cerebral metastatic lesion from malignant melanoma and clinical parameters characteristic of tumor behavior. They classified the metastases from melanomas to their size: smaller than 1 cm (group A), between 1 and 4 cm (group B), and bigger than 4 cm (group C), in order to assess the clinical course of the disease and predict the response to treatment. Group B lesions are the most common, independent of the site of the primary tumor, except for patients with rectal melanoma. Group C metastases are the least common and are usually solitary. Asymptomatic patients usually have group A metastases, whereas those with nonspecific complaints or behavioural changes usually have group B metastases. Solitary lesions usually belong to groups B or C, whereas multiple lesions belong mainly to groups A or B [3].

## 3. Epidemiology

CNS metastases occur in 10 to 40% of melanoma patients in clinical studies and up to 90% in autopsy studies [1]. In 15% to 20% of these patients, the CNS is the first site of relapse. In 41% a second organ is involved, and in 20% three organs are involved [4–7]. 

The cumulative risk at 5 years for patients with melanoma to develop CNS metastases corresponds to about 7% [8]. Fifty-eight percent of the patients with intracranial metastasis in malignant melanoma are male and 42% were female [9]. Brain metastases account for 20–54% of reported deaths from melanoma [8]. Malignant melanoma represents the third most common cause for central nervous system metastases after breast and lung cancer [10]. Whereas breast, lung, and kidney metastases are predominantly solitaire, malignant melanoma metastasizes often in a multiple way. Nevertheless, only about 5% of the patients with multiple melanoma metastases have more than five intracerebral metastatical lesions. 

Seventy-one percent of the primary lesions are invasive lesions with mean greater than thickness of 3.5 mm. Nevertheless, the studies have showed a significative prevalence of small and well-circumscribed lesions at surgical aspect. There are also observed indolent lesions with a long time of evolution [9].

The case of disseminated carcinomatous cell spreading throughout the brain is called “military metastases” or “carcinomatous encephalitis.” This condition is very rare and correlated with a poor prognosis. Most patients with advanced military metastases will have widespread extracranial disease, but the majority will die from intracerebral spread.

## 4. Risk Factors

Risk factors for central nervous system (CNS) metastases among patients with coetaneous malignant melanoma are: male, head and neck or oral primary lesion, presence of visceral metastases, mainly lung, primary tumor thickness and ulceration of primary lesion. Age and race are not significant factors [11, 12].

## 5. Molecular Biology

### 5.1. Endothelial Cell Heparanases

 One of the many features of the malignant melanoma phenotype, in vitro and in vivo, is the elevated heparanase production and activity, which confers the capacity of degrading the subendothelial matrix produced by endothelial cell monolayer cultures. Supra-additive levels of heparanase activity are found when brain endothelial cells are coincubated with brain-metastatical melanoma cells in equicellular amounts [13]. 

Murine and human brain-metastatical melanoma cells solubilize sulfated matrix proteoglycans at levels significantly higher than their parental lines. Sulfated matrix proteoglycans are rich in heparan sulfate (HSPGs), with minor amounts of chondroitin and dermatan sulfates. The pattern of HSPG degradation by brain-metastatical melanoma cells differs from that of less metastatical parental cells or cells metastatical to organs other than the brain. Cooperative interactions between heparanases from tumor and endothelial sources are suggested to be a significative mechanism in the invasion process.

### 5.2. Neurotrophins

The brain is a unique microenvironment enclosed by the skull and maintaining a highly regulated vascular transport barrier. To metastasize to the brain, malignant tumor cells must attach to microvessel endothelial cells, invade the blood-brain barrier (BBB), and respond to brain survival and growth factors. 

Neurotrophins (NTs) are important in brain invasion because they stimulate this process. In brain-metastatic melanoma cells, NTs can promote invasion by enhancing the production of extracellular matrix degradative enzymes such as heparanase, an enzyme capable of locally destroying both the extracellular matrix and the basement membrane of the BBB. Melanoma cell lines exhibiting low ability to form brain metastases express low-affinity neurotrophin receptor p75NTR in relation to their brain-metastatic potentials. Presence of functional TrkC, the putative receptor for the invasion-promoting neurotrophin NT-3, is also expressed in brain-metastatic potential cells.

Brain-metastatic melanoma cells can also produce autocrine factors and inhibitors that influence their growth, invasion, and survival in the brain. Synthesis of these factors may influence NTs production by brain cells adjacent to the neoplastic invasion front, such as oligodendrocytes and astrocytes. In brain biopsies, increased amounts of nerve growth factor (NGF) and NT-3 were observed in tumor-adjacent tissues at the invasion front of human melanoma tumors. Astrocytes are supposed to contribute to the brain-metastatic specifity of melanoma cells by producing NTs-regulated heparanase. Trophic, autocrine, and paracrine growth factors may, therefore, determine whether metastatical cells can successfully invade, colonize, and grow in the central nervous system [14].

### 5.3. Integrin alpha(v)beta(3)

Integrin alpha(v)beta(3) is a molecule of adhesion to the endothelium, and its expression on the metastatical pattern of human melanoma cells in the central nervous system (CNS) is already studied. Although it is predicted that the adhesion of tumor cells to endothelial cells plays a role in this phenomenon, tumor cell alpha(v)beta(3) integrand expression per se does not explain the difference in metastatic behavior in the CNS. Probably others yet unknown factors must be involved [15]. 

## 6. Signs and Symptoms

 Headache is the most common presenting symptom, but brain metastases should be suspected in all melanoma patients with new neurological findings [1]. In patients with brain metastasis, melanoma is one of the primaries cancers with the highest frequency of seizures, found in about one third of the patients [16]. Mean time interval between the initial diagnosis of melanoma and development of first symptoms of CNS metastases is 3.5 years [9].

### 6.1. Diagnosis

Brain metastases are clinically diagnosed in the majority of patients with metastatic melanoma. The time from diagnosis of the primary tumor to discovery of disease in the CNS is significantly longer for those who had group A lesions (metastases smaller than 1 cm), compared with those who had groups B (metastases between 1 and 1.4 cm) or C lesions (metastases bigger than 4 cm) [17, 18]. In patients whose disease had progressed to brain metastases, freedom from such metastases decreases logarithmically with time from initial presentation. This suggests a random distribution of progression rates with a mean time of 2.5 years between diagnosis and development of intracranial metastases [19]. 

#### 6.1.1. Computed Tomographic (CT)

All patient with new neurological signs and a previous primary melanoma lesion must be investigated. CT scan finds solitary lesions in 54.2% and multiple lesions in 45.8% of patients with malignant melanoma cerebral metastasis. Eighty-four percent of the solitary lesions are located in the cerebral hemispheres with 62.5% of these in the frontal region.

Seventy-five percent of the CNS metastatic melanoma lesions appear on noncontrast study as increased density; 22% are hypodense, and 3% are isodense. All lesions show contrast enhancement, usually appearing as a homogeneous nodular or ring pattern [20].

#### 6.1.2. Magnetic Resonance Images (MRI)

**Figure 1:**
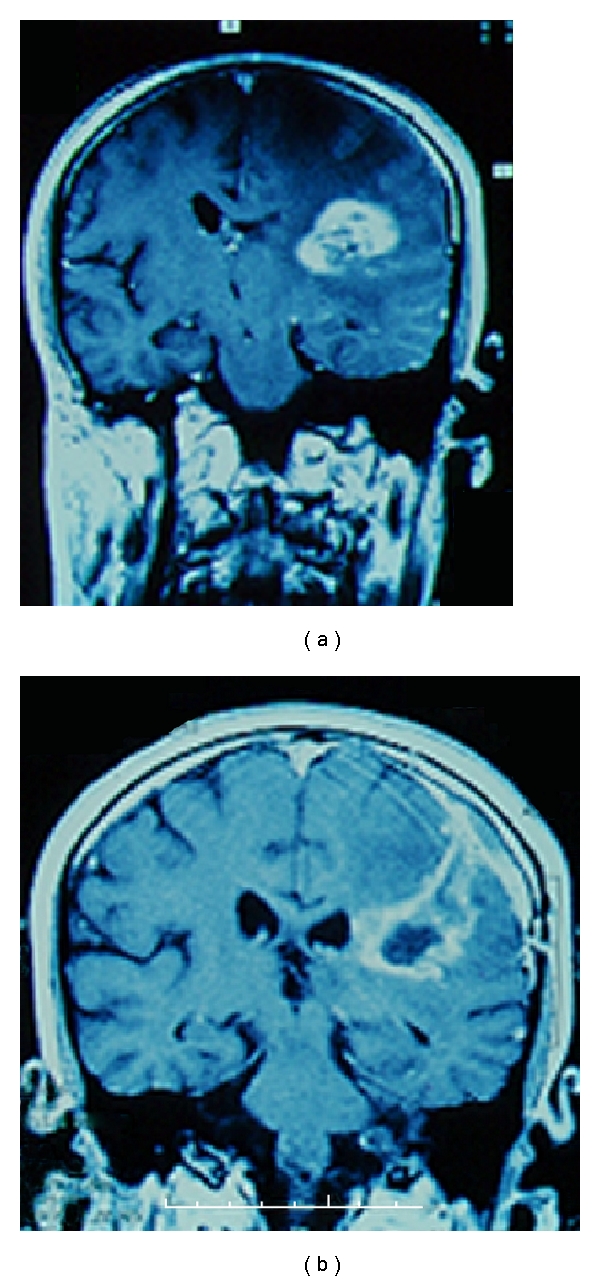
(a) axial T1-Weighted MRI after injection of Gadolinium, demonstrating an intraparietal lesion with heterogeneous appearance, presenting hypersignal intercalted with necrotic areas. (b) The same lesion in a coronal view.

MRI is the best diagnostic technique for detecting CNS metastases [1] (Figure 1). However, large, solitary, necrotic metastases can be indistinguishable from high-grade astrocytomas. Using conventional MRI, the demonstration of an elevated rCBV (relative cerebral blood volume CBV—the ratio between the normal adjacent and the pathological area) may suggest a hypervascular lesion such as renal carcinoma or melanoma [21].

**Figure 2:**
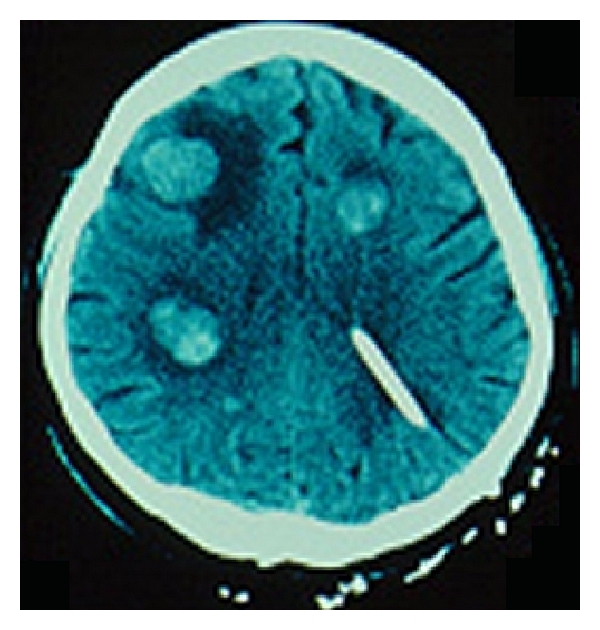
CT demonstrating multiple melanoma metastatic lesions. Observe the typical hyperintense signal and the corticosubcortical localization. Note also the presence of an external derivation catheter in left ventricle as an attempt to alleviate intracranial hypertension.

Most commonly, CNS melanoma metastasis appears in MRI as hyperintense (Figure 2) on T1-weighted images and hypointense on T2-weighted images. Hemorrhage in the lesion may have a greater influence on this unique appearance than does melanin. The increased tissue sensitivity of MRI allow for 22% of patients greater lesion detection than did CT [22].

#### 6.1.3. Optical Coherence Tomography (OCT)

Intraoperative identification of brain tumors and tumor margins has been limited by either the resolution of the *in vivo* imaging technique or the time required to obtain histological specimens. 

Optical coherence tomography (OCT) is a new, noncontact, high-speed and high-resolution, real-time, intraoperative imaging technique, capable of resolutions on micrometer scale, which has been used to identify intracortical melanomas. OCT is analogous to ultrasound B-mode imaging, except that reflections of infrared light, rather than sound, are detected. OCT uses inherent tissue contrast, rather than enhancement with dyes, to differentiate tissue types. The compact, fiber-optic-based design is readily integrated with surgical instruments.

Two-dimensional images show increased optical backscatter from regions of metastatic melanoma tumors, which are quantitatively used to determine the tumor margin. The images correlate well with the histological findings. Three-dimensional reconstructions reveal regions of tumor penetrating normal cortex and can be re-cut at arbitrary planes. Subsurface cerebral vascular structures can be identified and are then avoided during the surgery. The Literature reports suggest that OCT can effectively differentiate normal cortex from intracortical melanoma based on variations in optical backscatter [23]. High-resolution and high-speed imaging capabilities of OCT may permit the intraoperative identification of tumor and more precise localization of tumor margins.

#### 6.1.4. Positron Emission Tomography (PET)

Detection and diagnosis of human malignant melanoma by Positron Emission Tomography (PET) using 18F-10B-L-BPA, a specific melanogenesis-seeking compound, has been developed. This resulted in a novel, highly effective methodology for the selective three dimensional imaging of metastatic malignant melanomas, and for accurate determination of 18F-10B concentration in the tumor and surrounding tissue, providing almost all diagnostic information necessary for complete noninvasive radiation dose planning in the treatment of malignant melanoma [24].

#### 6.1.5. Iofetamine (I 123) Cintilography

The Literature data show that iofetamine (I 123) cintilography can be a good method to differential diagnostic in patients with risk for metastatic melanoma lesions. In patients with metastatic melanoma lesions, cintilography shows increased uptake of iofetamine (I 123). Studies suggest that certain brain tumors such as melanoma are capable of selectively binding iofetamine I 123 because of specific chemical properties of this radiopharmaceutical compound. The sensibility of this method is 80% and the specificity 97.8% [25].

#### 6.1.6. Immunohistochemistry

Intermediate filament keratin is regarded as a good marker for epithelial and mesothelial tumors. In the intracranial and intraspinal spaces keratin has been demonstrated only in the endocrine cells of the adenohypophysis, squamous epithelial islands in the pars tuberalis of the hypophysis, and in the choroid plexus epithelium. Since gliomas and meningiomas do not express keratin, immunohistochemistry essay for keratin provide an additional help for differentiating between metastatic melanomas and primary central nervous system tumors [8]. 

#### 6.1.7. Monoclonal Antibody Immunocytology

The addition of monoclonal antibody immunocytology to conventional techniques significantly improves the sensitivity of CSF cytology. This is particularly useful in the diagnosis of “Neoplastic meningitis,” also called metastatic meningeal melanomatosis (MMM) a complication of malignant melanoma disease whose diagnosis rests on the demonstration of malignant cells within the CSF [26].

The putative CSF tumor markers, fibronectin, and beta 2-microglobulin, are elevated significantly in MMM (metastatic meningeal melanomatosis) but not in patients with solid cerebral metastases. A prominent increase in the IgM index, which reflects intrathecal B-cell stimulation, and rise of IgG index, interleukin-6, and tumor necrosis factor-alpha in MMM patients provide preliminary evidence for a local intrathecal immune response triggered by melanoma cell invasion of the subarachnoid space [27].

#### 6.1.8. Transcriptase-Polymerase Chain Reaction (RT-PCR)

Diagnosis of melanoma CNS metastases typically is made following the onset of clinical symptoms. Thus, more sensitive diagnostic approaches are needed to identify subclinical CNS metastases. Currently, standard cytological analysis of the cerebrospinal fluid (CSF) is limited by its poor sensitivity. A more sensitive assay was therefore developed using multiple reverse transcriptase-polymerase chain reaction (RT-PCR) markers.

The CSF is collected and assessed by RT-PCR for three known melanoma-associated markers (MAGE-3, MART-1, and tyrosinase) to detect occult metastatic melanoma cells in the CSF. The correlation between CSF RT-PCR positivity of MART-1 and/or MAGE-3 and the development of CNS metastases is significant. Of the patients with positive CSF RT-PCR markers, 41% have either positive MRI and/or positive RT-PCR results [28].

### 6.2. Treatment

The optimal treatment of melanoma patients with CNS metastases depends on each situation. Often surgery, radiosurgery, whole brain radiotherapy and chemotherapy are used in combination to obtain longer remissions and optimal symptom relieve [1]. Patients with multiple metastases usually receive whole brain irradiation (WBI) [5]. Patients with limited CNS metastases and widespread systemic disease can achieve prolonged survival with targeted treatment of CNS lesions and aggressive systemic therapy—chemotherapy.

Gamma Knife radiosurgery or surgical resection of CNS disease prior to chemotherapy improves survival versus delayed treatment in patients melanoma with melanoma brain metastases [4]. Radiotherapy is recommended to those cases where total resection were not possible and the concomitant use of corticoid pulse therapy is recommended in order to reduce peritumoral edema and mass tumor effect.

#### 6.2.1. Surgical Resection

**Figure 3:**
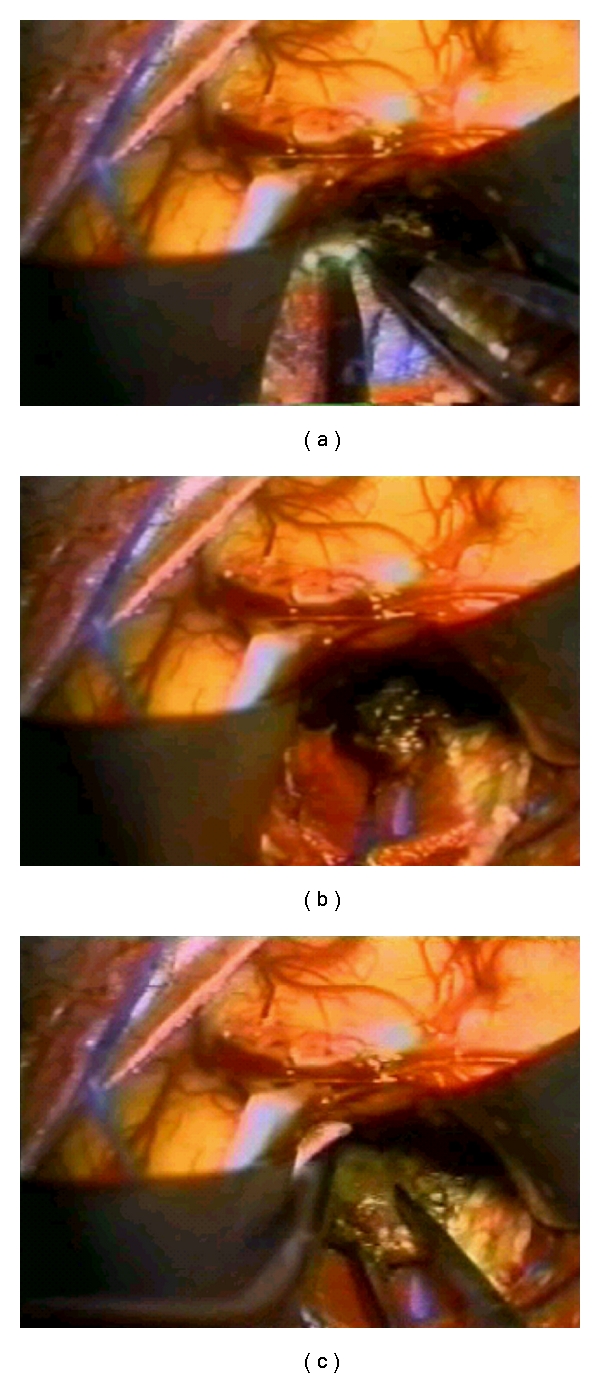
Sequence of surgical photos showing intraparietal exposition of a CNS metastatical melanoma lesion and its surgical excision.

Surgery of an isolated metastasis can lead to a long survival but brain lesions are frequently numerous and associated with an extracerebral diffusion (Figure 3). Complete surgical resection of intracranial melanoma metastatic lesions results in a mean survival period of 10.3 months. Patients with primary lesions of the head and neck have lowest mean survival, about 3.3 months, whereas those whose primary sites are unknown have the longest mean survival, approximately 7.5 months. General 1- and 2-year survival rates are 9% and 3%, respectively [29].

#### 6.2.2. Whole Brain Irradiation (WBI)

 Duration and quality of survival depend on the extent of metastatic disease and response to treatment. Treatment goals with WBI are palliation of symptoms and prolongation of life. Although brain metastases may be treated with surgery and/or stereotactic radiosurgery (SRS) when disease is limited to three lesions, treatment for patients with large or multiple metastases is limited to WBI. 

While formal response and survival analyses of the impact of WBI in melanoma metastases have not been reported, the estimated mean survival time for unselected patients with CNS metastases is only 2 to 4 months, with 1-year survival rates of less than 13%. This rates prevent the use of WBI as single therapeutic option. In a selected population of patients with limited CNS involvement, surgical resection alone or in combination with WBI appears to prolong mean survival. Overall survival is significantly improved in patients with multiple metastasis who receive adjunctive cranial irradiation versus those who had surgery alone [30]. 

Therefore, adjunctive cranial irradiation is justified for melanoma patients who undergo surgical therapy for solitary brain metastases. Survival in patients presenting with solitary brain metastases is improved by a reduction of relapse in the brain as a component of failure by combined surgery and irradiation. In these patients survival will depend basically upon control of systemic disease Some authors even suggest the prophylactic whole brain irradiation for patients with melanoma that present high risk of metastases, once CNS metastases are sometimes the sole site of clinical relapse, and are frequently disabling [31].

#### 6.2.3. Accelerated Irradiation Regimens

 In accelerated irradiation regimens, the total tumor dose varies from 3000 to 4800 rad, and the overall treatment time from 1 to 2 weeks. This more aggressive form of treatment has demonstrated no significant improvement in the results from accelerated fractionation in the treatment of intracranial metastases. 

The result of the radiotherapy treatment did not depend on the site of the primary lesion, number of intracranial metastases, total dose, or the dose perfraction. There are, however, two subgroups not mutually exclusive, that benefit significantly from the accelerated fractionation: patients who had a complete resection of brain metastases, and those having no detectable extracranial metastases at the time of their treatment for intracranial metastases [19].

#### 6.2.4. Chemotherapy

Although in larger metastases the blood-brain barrier is leaky, lesions are resistant to many chemotherapeutic drugs. While systemic therapy for metastatic melanoma produces objective responses in 15% to 50% of patients, available drugs do not penetrate well into the CNS, and these patients rarely benefit from systemic therapy [6].

**(a) Dacarbazine**

Dacarbazine (DTIC) gives a mean response rate of 21% on visceral localizations but does not cross the blood brain barrier (BBB). Biological response modifiers like Interleukin 2 (Il2) leads to a 25% response rate in disseminated melanoma [29].

**(b) PCNU**

The results indicate that systemic PCNU is unlikely to be more effective than other currently used chemotherapy in patients with malignant melanoma and CNS metastases [32]. Moreover, treatment with PCNU presents frequently some acute complications like granulocytopenia or thrombocytopenia.

**(c) Temozolomide**

Temozolomide (TMZ) is a novel oral alkylating similar to dacarbazine (the most active single agent in primary melanoma) that have 100% oral bioavailability and considerable penetration of CNS tissue. TMZ has broad preclinical antitumoral activity that in melanoma is comparable to that of dacarbazine.Sites of remission of metastatic melanomas treated with temozol*o*mide include brain, lung, liver, lymph nodes and muscle. Patients tolerate treatment with termozolomide well and usually no dose reduction is necessary. However some few patients may present complications due to severe leucopoenia and thrombocytopenia (WHO grade 3 and 4). Thus, termozolomide represents a safe treatment option in patients with metastatic melanoma and poor prognosis [33].

**(d) Temozolomide Combinations**

Temozolomide (TMZ) may also be used in combination with interferon alpha-2b (IFN-alpha2b). The most common adverse events during the use of this association are fatigue, fever, nausea/emesis, anxiety, and diarrhea. Most toxicity is mild to moderate in severity. The primary dose-limiting toxicity is thrombocytopenia. The maximum tolerated dose is either TMZ 150 mg/m(2) plus IFN-alpha2b 7.5 MIU/m(2) or TMZ 200 mg/m(2) plus IFN-alpha2b 5.0 MIU/m(2). The pharmacokinetics of TMZ is not affected by coadministration of IFN-alpha2b [33, 34]. The combination of TMZ and whole brain irradiation (WBI) was studied in patients with CNS metastatic malignant melanoma, and it was found that, although TMZ can be safely administered with WBI, the combination has limited antitumor activity [35]. Another option for the chemotherapy in patients with metastatic melanoma is the use of termozolomide (TMZ) p.o. followed by subcutaneous (s.c.) low-dose interleukin-2 (IL2), granulocyte-monocyte colony stimulating factor (GM-CSF), and interferon-alpha 2b (IFN alpha). The overall objective response rate Is 31%. Responses occur in all disease sites including the central nervous system (CNS) [36]. The main toxicity of this combined chemotherapy is the flu-like syndrome and transient liver function disturbances. The mean overall survival with temozol*o*mide reported is 7 months since beginning of therapy.

**(e) Fotemustine (Muphoran)**

Fotemustine (muphoran), a new amino acid-linked nitrosourea, can give a response rate up to 28.2% in patients with cerebral metastases, and the increased survival of responding patients is significant [6, 29]. All responders to fotemustine have mainly cortical, group A (lesion smaller than 1 cm), or group B lesions (lesion s between 1.1 and 4 cm). Patients with group C metastasis (lesion bigger than 4 cm) or leptomeningeal spread do not respond to fotemustine.

**(f) Intracarotid Cisplatin**

Intracarotid cisplatin-based chemotherapy may be useful for palliation in selected patients with malignant melanoma and CNS metastases. Intracarotid cisplatin 40–75mg/m^2^ can be administered alone, with 1,3-bis(2-Chloroethyl)-1-nitrosourea (BCNU) or with bleomycin. Thirty percent of the patients have objective improvement in CT scans and about 13% of them have stabilization of disease. The median time to tumor progression for responding patients is 20 weeks. Neurological and retinal toxicity are potential complications of this therapy [37].

**(g) Interferon**

Interferon apparently is inactive against melanoma brain metastases, but does cause CNS symptoms [38].

#### 6.2.5. Stereotactic Radiosurgery

More recently, stereotactic radiosurgery (SRS) has been used as an effective local treatment for patients with CNS melanoma metastases. In several retrospective reports, treatment with SRS alone or in combination with whole brain irradiation (WBI) has demonstrated to prolong mean survival [39]. As happens with the treatment with WBI alone, most of the patients die from progressive extracranial disease with locally controlled CNS disease [6, 40].

#### 6.2.6. Gamma Knife Surgery

Gamma knife surgery (GKS) is effective in treating melanoma metastases in the brain. It appears that the radiobiology of a single high dose overcomes the radioresistance barrier, yielding better results than fractionated radiation. Twenty-four percent of the lesions treated with GKS disappear, 35% shrink, 23% remain unchanged, and 18% increasing size. No undue radiation-induced changes are observed in the surrounding brain. No deaths or neurological morbidity related to GKS is observed. The mean survival time calculated is 10.4 months from the time of treatment with GKS. Solitary brain lesions and lack of visceral metastases are statistically predictive of a better prognosis [12].

#### 6.2.7. Boron Neutron Capture Therapy (BNCT)

Boron neutron capture therapy (BNCT) is yet an incipient therapy method that has shown good results in rats experiments, mainly if combined by either Cereport (RMP-7) mediated modulation of blood-brain barrier (BBB) permeability or hyperosmotic mannitol-induced BBB disruption using borono-phenylalanine (BPA) as the capture agent. Optimizing the delivery of BPA by means of intracarotid injection combined with opening the BBB by infusing Cereport or a hyperosmotic solution of mannitol significantly enhance survival times and produce long-term cures in animals with CNS melanoma metastasis. These observations are relevant to future clinical studies using BNCT for the treatment of intracerebral melanoma [41].

#### 6.2.8. Prognosis

Prognosis for patients with melanoma brain metastasis is still poor with a mean survival time of 6 months after diagnosis [1, 2, 5, 42]. Mean survival in patients treated with chemotherapy is approximately 8.3 months—1 year survival of 41%. Surgical resection allows mean survival rates of 10,3 months. Estimated mean survival time for patients with CNS melanoma metastases treated with whole brain irradiation is only 2 to 4 months. Mean survival time for patients treated with Gamma knife radiosurgery is 10.4 months [36].

## 7. Conclusions

Most of the symptoms of CNS melanoma metastasis are unspecific and depend on localization of the lesion. CT scan is recommended as the primary exam in case of suspected cases. MRI must be used to study more precisely the characteristics of the lesions in order to embase the surgical plane. It is important that the clinician suspect of the possibility of CNS metastasis in all patients with new neurological signs and previous primary melanoma lesion. These patients deserve a careful clinical analysis and further exams for investigation (Table 1).

Optimal treatment of melanoma patients with CNS metastases depends on the objective situation. Often surgery, radiosurgery, whole brain radiotherapy and chemotherapy are used in combination to obtain longer remissions and optimal symptom relieve (Table 2). Patients with multiple metastases usually receive whole brain irradiation (WBI). Patients with limited CNS metastases and widespread systemic disease can achieve prolonged survival with targeted treatment of CNS lesions and aggressive systemic chemotherapy, when clinical conditions of patient allow. Stereotactic radiosurgery is recommended to those cases where total resection are not possible and, in this case, the concomitant use of corticoid pulse therapy may be useful in order to reduce peritumoral edema and mass tumor effect. We highly recommend “New Brain Metastases Treatment Guidelines” issued by the American Association of Neurological Surgeons & the Congress of Neurological Surgeons published in the Journal of Neuro-Oncology in 2009. It deals with specific issues regarding the level of evidence of chemotherapy, radiosurgery, surgical resection, whole brain radiotherapy, use of corticosteroids, and anticonvulsants in the treatment of brain metastases.

## Figures and Tables

**Table 1:** List of available diagnostic methods, their specific utility and Results which suggeest CNS melanoma metastasis.

Diagnostic methods	Suggestive result	Specific utility
Computed tomography	Hyperdense lesion	Primary exam

Magnectic resonance Images	Hyperintense lesion on T1 and hypointense lesion on T2-weighted images	More detailed exam

Optical coherence tomography	Increased optical backscatter	Intraoperatory exam

Positron emission tomography	High captation of compound 18F-10B-LBPA	Noninvasive radiation dose planning

Cintilography	Increased uptake of iofetamine	High sensible method

Immunohistochemistry	Finding of intermediate filament keratin	Differentiating between metastatic melanomas and primary central nervous system tumors

Monoclonal antibody	Raise of fibronectin, beta 2-microglobulin	Diagnosis of metastatic meningeal melanomatosis

Immunocytology	IgM and IgG index, IL-6 and TNF-alpha	Diagnosis of metastatic meningeal melanomatosis

Transcriptase-polymerase chain reaction (PCR)	Detection of melanoma-associated markers (MAGE-3, MART-1, and tyrosinase)	Diagnosis of subclinical CNS metastases

**Table 2:** List of therapeutic options mean survival and specific indications of each method.

Treatment modality	Mean Survival	Indications
Surgical resection	10,3 months	Limited (up to three) CNS metastases and widespread systemic disease
Whole brain irradiation	2–4 months	Large or multiple metastases
Chemotherapy	8,3 months	Widespread systemic disease and multiple CNS metastasis
Stereotactic Radiosurgery	1 year	Small and solitary lesions
Gamma knife surgery	10,4 months	Small and solitary lesions
Boron neutron capture therapy	Still in animal research phase	—
